# Effect of Changing Match Format from Halves to Quarters on the Performance Characteristics of Male University Field Hockey Players

**DOI:** 10.3390/s21165490

**Published:** 2021-08-15

**Authors:** Elliot P. Lam, Caroline D. Sunderland, John G. Morris, Laura-Anne M. Furlong, Barry S. Mason, Laura A. Barrett

**Affiliations:** 1 School of Sport, Exercise and Health Sciences, Loughborough University, Loughborough LE11 3TU, UK; E.Lam@lboro.ac.uk (E.P.L.); L.A.M.Furlong@lboro.ac.uk (L.-A.M.F.); barry.mason@gbwr.org.uk (B.S.M.); L.A.Barrett@lboro.ac.uk (L.A.B.); 2 Department of Sport Science, School of Science and Technology, Nottingham Trent University, Nottingham NG11 8NS, UK; john.morris@ntu.ac.uk; 3 Department of Physical Education and Sport Sciences, University of Limerick, V94 T9PX Limerick, Ireland

**Keywords:** GPS, field hockey, performance characteristics

## Abstract

The study examined whether the performance characteristics of male university field hockey players differed when the match format was 2 × 35 min halves compared to 2 × 2 × 17.5 min quarters. Thirty-five male university field hockey players (age 21.2 ± 3.0 years, height 1.81 ± 0.07 m, body mass 75.1 ± 8.9 kg), competing at national level in the UK, were monitored over 52 matches played across the 2018–2019 (2 × 35 min halves) and 2019–2020 (2 × 2 × 17.5 min quarters) seasons using 15 Hz Global Positioning System units and heart rate monitors. Total distance, high-speed running distance (≥15.5 km·h^−1^), accelerations (≥2 m·s^−1^), decelerations (≤−2 m·s^−1^), average heart rate and percentage of time spent at >85% of maximum heart rate were recorded during both match formats. Two-level random intercept hierarchal models (Match—level 1, Player—level 2) suggested that the change in format from 2 × 35 min halves (2018–2019 season) to 2 × 2 × 17.5 min quarters (2019–2020 season) resulted in a reduction in total distance and high-speed running distance completed during a match (by 221 m and 120 m, respectively, both *p* < 0.001). As no significant cross-level interactions were observed (between season and half), the change from 35 min halves to 17.5 min quarters did not attenuate the reduced physical performance evident during the second half of matches (total distance: −235 m less in second half; high-speed running distance: −70 m less in second half; both *p* < 0.001). Overall, the findings suggest that the change in match format did alter the performance characteristics of male university field hockey players, but the quarter format actually reduced the total distance and high-speed running distance completed during matches, and did not attenuate the reduction in performance seen during the second half of matches.

## 1. Introduction

The effective prescription of training programmes is reliant upon a comprehensive understanding of the performance characteristics of the particular sport [1,2]. In a team sport such as field hockey the assessment of performance characteristics (such as the total running distance completed, the frequency of sprinting during matches or the heart rate response) has been facilitated by the emergence of Global Positioning System (GPS) technology, integrated with accelerometry and heart rate telemetry [3,4]. Previous research examining performance characteristics in field hockey has reported that male international players run > 90% of the total distance travelled (~5000–10,000 m) at low-to-moderate running speeds, but the frequent performance of sprints, accelerations and decelerations emphasise the intermittent, high-intensity nature of the sport [5,6,7,8,9].

However, the utility of previously published research findings about the performance characteristics of field hockey may be limited for current players and practitioners given the changes made in 2014 to the format of international matches by the International Hockey Federation (FIH) from 2 × 35 min halves to 2 × 2 × 15 min quarters, with overall match duration changing from 70 to 60 min, respectively. Forty-second time outs for penalty corners and when a goal was scored were also introduced. These rule changes were introduced to improve the flow and intensity of the game, facilitating an improved fan experience by providing more opportunities for commentator analysis and replays between plays and during a match [10,11]. The new match format also allows players and coaches additional opportunities to rehydrate and alter strategy during a match with four quarters.

England Hockey introduced a unique variation of this two-quarters-per-half format for national-level competition in 2019, restructuring match duration from 2 × 35 min halves to 2 × 2 × 17.5 min quarters, with no time outs for penalty corners or goals scored. In previous research into field hockey players, based on a 2 × 35 min half format, a decrease in the total distance and a 1.6–15% reduction in high-speed running distance (HSR) completed in the second half of matches was observed, with these decrements in performance from first to second half being attributed to fatigue [4,6,7,12]. In contrast, a recent investigation involving male international field hockey players, and a match format of 2 × 2 × 15 min quarters, found no evidence of a decrement in the high-speed running distance completed by players in each quarter of a match [13]. Morencos and colleagues [14] also found no evidence of a decrement in high-speed running distance completed by Spanish national league players when the match format was 2 × 2 × 15 min quarters. Therefore, the data that have been published so far do indeed suggest that a 2 × 2 × quarter format may attenuate some of the variation in some performance characteristics evident when matches are structured as two halves. However, the studies of the performance characteristics of field hockey players when matches were played in quarters by Ihsan and colleagues [13] and Morencos and colleagues [14] were based on data collected over one season or a single tournament, and so a direct comparison between the match formats of 2 × halves and 2 × 2 × quarters was not made.

To date, only McMahon and Kennedy [11] have directly compared performance characteristics in field hockey matches where the format was 2 × 35 min halves compared with 2 × 2 × 15 min quarters. Employing effect size analysis (*d*) to assess female international players over two consecutive seasons, the study found small–moderate increases in distance run per minute (halves vs. quarters: 109.1 ± 12.6 vs. 113.3 ± 13.5 m·min^−1^; *d* = 0.57) and the average high-speed running distance (distance covered at speeds greater than 3.08 m·s^−1^/time spent at speeds greater than 3.08 m·s^−1^) (halves vs. quarters: 296.3 ± 7.1 vs. 298.2 ± 6.6 m·min^−1^; *d* = 0.28) with the quarter format, and concluded that some performance characteristics of field hockey were indeed greater when matches were structured as 2 × 2 × quarters rather than 2 × halves. However, the study was focused on seasonal and positional comparisons of performance characteristics across whole matches, and did not examine how changes to match format influenced the decrement in many performance characteristics typically seen in the second half of field hockey matches.

Therefore, the aim of the current study was to determine whether the change in match format from 2 × 35 min halves to 2 × 2 × 17.5 min quarters altered the performance characteristics (total distance, high-speed running, accelerations, decelerations, heart rate responses) of male university field hockey players across a match and between halves within a match. It was hypothesised that an improvement in performance characteristics would be seen in the quarter format compared to the halve format due to the additional recovery between quarters.

## 2. Materials and Methods

### 2.1. Experimental Approach to the Problem

A longitudinal study design was used to examine the performance characteristics (total running distance, high-speed running distance, number of accelerations and decelerations, heart rate responses; see detailed descriptions below) of male university field hockey players during matches structured as 2 × 35 min halves or as 2 × 2 × 17.5 min quarters. All matches were played in two national league competitions, namely the England Hockey League Division One North (EHL) and the British Universities and Colleges Sport Hockey Premier North A League (BUCS). Matches were played in the United Kingdom (UK) under temperate conditions (temperature 7.8 ± 3.1 °C, relative humidity 83 ± 9%) over two consecutive seasons (2018–2019, 2019–2020). Matches comprised 2 × 35 min halves (~10 min half-time interval) during the 2018–2019 season, whilst matches played in the 2019–2020 season comprised 2 × 2 × 17.5 min quarters (~2 min quarter-time and 7 min half-time intervals). Performance characteristics were examined using 15 Hz Global Positioning System (GPS) data (SPI HPU, GPSports, Canberra, Australia) and heart rate measurements (Polar T31, Polar Electro, Kempele, Finland).

### 2.2. Participants

Thirty-five male field hockey players (age 21.2 ± 3.0 years, height 1.81 ± 0.07 m, body mass 75.1 ± 8.9 kg) competing for the 1st team of a university field hockey club volunteered to participate in the study. Seventeen of the players were junior internationals (competing at under-18 or under-21 level, or for Great Britain’s elite development squad). Data on performance characteristics were available for twenty-two outfield players from the 2018–2019 season (primary playing positions: defenders (*n* = 6), midfielders (*n* = 8) and forwards (*n* = 8)). Data on performance characteristics were available for twenty-two outfield players from the 2019–2020 season (primary playing positions: defenders (*n* = 7), midfielders (*n* = 7) and forwards (*n* = 8)). Nine players were present for both seasons and goalkeepers were excluded from the study. Twenty-eight matches (EHL = 16, BUCS = 12) were analysed in the 2018–2019 season and twenty-four matches (EHL = 16, BUCS = 8) were analysed in the 2019–2020 season, yielding 604 individual player match files. Written and verbal communication of the aims, procedures and risks of the current research were provided to all players. Players provided opt-out consent prior to their involvement in the study. Ethical approval was granted by an institutional university ethics committee (Ref No: R19-PO19). 

### 2.3. Procedures

Performance characteristics during competitive matches was quantified using 15 Hz GPS units (SPI HPU, GPSports, Canberra, Australia). Players wore a specialised, elasticated harness underneath their playing shirt that positioned the GPS unit on the upper back (T1–T6). GPS units were activated outside and left stationary to enable an accurate number of satellite signals to be obtained (>6 satellites). Once the satellite signals had been obtained, players inserted their GPS units into their harnesses before every pre-match warm-up. The mean satellite signal strength was 9 ± 1 and horizontal dilution of precision was 0.48 ± 0.04, where values less than 1 represent an ideal positional accuracy [15]. Heart rate was recorded using a monitor (Polar Electro T31, Kempele, Finland). Players secured their monitor onto their skin using an elasticated chest strap, just below the sternum. During matches, heart rate was continuously recorded and data logged simultaneously with the external data collected by the GPS units. Players were assigned the same GPS unit and heart rate monitor for every match throughout the study period to avoid interunit error.

Post-match, the data on the GPS units were downloaded and processed on a laptop computer containing the manufacturer’s analysis software (GPSports Team AMS, v.R1 2019, Canberra, Australia). Each GPS match file was trimmed to only include time periods when a player was on the pitch, as described by White and MacFarlane [8]. The time-on-pitch data from each individual player match file were exported from the analysis software into customised Excel spreadsheets (Office 365, Microsoft Office, Redmond, WA). GPS and heart rate data from each individual player match file were used to calculate performance characteristics for the whole match and for the first and second halves of matches in the 2018–2019 and 2019–2020 seasons. In the 2019–2020 season the data from the first and second quarters of matches, and from the third and fourth quarters of matches, were combined to give equivalent first and second half data, respectively.

The following GPS variables were used to examine the performance characteristics of the players in the current study: total running distance (m); high-speed running distance (HSR; m ≥ 15.5 km·h^−1^); accelerations (n; ≥2 m·s^−2^ lasting over 1 s) and decelerations (n; ≤−2 m·s^−2^ lasting over 1 s); distance (total) per minute (m·min^−1^); high-speed running percentage (% of total distance); accelerations per minute (n·min^−1^); and decelerations per minute (n·min^−1^). The absolute high-speed running threshold chosen has been used in previous field hockey research [16,17], based upon the original speed categories defined by Bangsbo and colleagues [18] and adapted from the sport-specific GPS velocity zones calculated for male field hockey [19]. The following heart rate variables were also used to examine the performance characteristics of the players in the current study: average heart rate (HR_AVE_; b·min^−1^); percentage of maximum heart rate (%HR_MAX_); peak heart rate (HR_PEAK_; b·min^−1^); peak heart rate as a percentage of maximum heart rate (HR_PEAK_; %HR_MAX_); duration (min) of total time spent at a zone exceeding 85% of maximum heart rate (Time spent > 85% HR_MAX_ (min)); percentage (%) of total time spent at a zone exceeding 85% of maximum heart rate (Percentage time > 85% HR_MAX_ (%)). Maximum heart rate was individually determined from the highest recorded value during fitness testing within a season or, if higher, a maximal value obtained during matches. Before inclusion in the modelling analysis (see detailed description below), all first and second half performance characteristics data were normalised to 35-min equivalent time (i.e., data from the 2018–2019 season were normalised to 35 min by dividing the actual performance characteristic value by time-on-pitch, and then multiplying by 35); data from the 2019–2020 season were normalised to 17.5 min quarters and then combined as described above.

### 2.4. Statistical Analysis

The ‘raw’ (relating just to actual time-on-pitch) Global Positioning System (GPS) and heart rate data recorded from matches during the 2018–2019 (2 × 35 min halves) and 2019–2020 season (2 × 2 × 17.5 min quarters) are presented as mean ± SD. An effect size (*d*) was used to examine between-season differences in time-on-pitch and was interpreted using the following thresholds: *d* < 0.2 = trivial, 0.2 ≤ *d* < 0.5 = small, 0.5 ≤ *d* < 0.8 = moderate and *d* ≥ 0.8 = large [20].

Given the hierarchal structure of the dataset (matches within players), multilevel modelling was used to examine if the performance characteristics of field hockey players changed when the match format was 2 × 35 min halves compared with 2 × 2 × 17.5 min quarters. Specialist software (MLwiN v.3.05, Bristol, UK) was used to fit two-level random intercept models (individual half match file (level 1; *n* = 1208) nested within player (level 2; *n* = 35)) to each continuous dependent variable (total distance (m), high-speed running distance (m), accelerations (n), decelerations (n), percentage of maximal heart rate (%), percentage of time spent at >85% of maximum heart rate (%)). Initially, a baseline/null model (model 1) was fitted giving the mean and SE of the intercept (fixed component), the unexplained between-player and between-file variance (random component) and the deviance value (−2*loglikelihood) that represented the goodness of the model fit. A series of fixed, explanatory covariates or contextual variables was progressively added to create more complex models in the following order, comparing a chosen reference category with its other categories: season (model 2; season 2018–2019 (reference) vs. 2019–2020); position (model 3; defenders (reference) vs. midfielders vs. forwards); half (model 4; 1st half (reference) vs. 2nd half); and a cross-level interaction between season and half (model 5; 2018–2019*1st half (reference) vs. 2019–2020*2nd half). After a fixed explanatory variable was added to the model, significant differences between the categories of each explanatory variable included within the new model were interpreted using the Wald statistic (parameter estimate/SE) [21]. To determine if the addition of an explanatory/contextual variable resulted in an improved overall model fit, it was necessary for the likelihood ratio test (deviance of model (as assessed by -2logliklihood) when explanatory variable was added—deviance of previous model) to show a reduction exceeding the critical value of a chi-squared distribution at the 5% level (*p* < 0.05). To assess if the change in match format from 2 × 35 min halves to 2 × 2 × 17.5 min quarters improved performance characteristics in the 2nd half of matches, an interaction effect between season and half was assessed within model 5 after controlling for the main effects of season, position and half.

## 3. Results

### 3.1. Performance Characteristics (Descriptive Data)

The performance characteristics associated with matches where the playing format was 2 × 35 min halves (2018–2019 season) or 2 × 2 × 17.5 min quarters (2019–2020 season) are presented in Table 1. The mean time-on-pitch across the two seasons was 49.7 ± 9.8 min. There was a 5% increase in time-on-pitch (*d* = 0.26) during the 2019–2020 season (when ‘halves’ comprised two quarters) compared to the 2018–2019 season (halves). The performance characteristics across the 2 × 2 × 17.5 min quarters played in the 2019–2020 season are presented in Table 2.

### 3.2. Performance Characteristics (Multilevel Models)

Table 3 shows the multilevel models examining the effects of season, position and half on normalised GPS and heart rate variables (model 4), during matches where the playing format was 2 x 35 min halves (2018–2019 season) or 2 × 2 × 17.5 min quarters (2019–2020 season).

The inclusion of season as an explanatory variable within the models suggests that total distance was reduced by 221 m (*p* < 0.001), high speed running distance was reduced by 120 m (*p* < 0.001) and percentage of time spent at > 85% maximum heart rate was reduced by 3.1% (*p* = 0.045) during halves played in the 2019–2020 season (when matches were structured as 2 × 2 × 17.5 min quarters). There were no between-season differences observed for the number of accelerations (*p* = 0.177) and number of decelerations (*p* = 0.280) completed in a half, or in the percentage of maximum heart rate (*p* = 0.09) maintained during a half.

The inclusion of position as an explanatory variable within the models suggests that defenders covered less total distance and less high-speed running distance, and performed fewer accelerations and decelerations in a half of match play compared to midfielders and forwards (*p* < 0.001; Table 3). Forwards also covered more high-speed running distance (*p* < 0.001) and completed a greater number of accelerations (*p* < 0.001) and decelerations (*p* = 0.027) in a half of match play than midfielders. No inter-positional differences were evident for the percentage of maximum heart rate maintained during a half (Midfielder vs. Defender, *p* = 0.262; Forward vs. Defender, *p* = 0.395) or in the percentage of time spent at >85% maximum heart rate (Midfielder vs. Defender, *p* = 0.277; Forward vs. Defender, *p* = 0.281).

The inclusion of half as an explanatory variable within the models suggests that total and high-speed running distance was less, the number of accelerations and decelerations fewer, the percentage of maximum heart rate maintained during a half was lower and the percentage of time spent at >85% maximum heart rate was less in the 2nd half of matches compared to the 1st half (*p* < 0.001).

**Figure 1:**
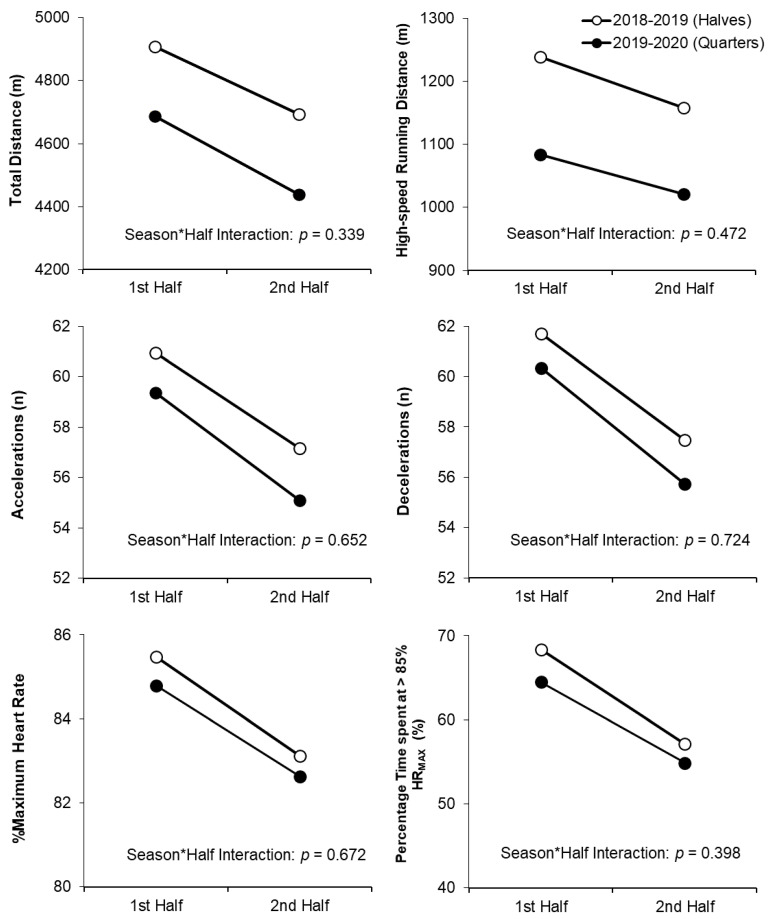
Mean values obtained from multilevel models of 1st and 2nd half match play data, from matches where the playing format was 2 × 35 min halves (2018–2019 season) or 2 × 2 × 17.5 min quarters (2019–2020 season). Dependent variables were normalised by extrapolating values to the full half playing time of 35 min. Percentage of time spent at >85% HR_MAX_ = percentage (%) of total time spent at a zone exceeding 85% of maximum heart rate. There were no cross-level interaction effects observed between season and half for the dependent variables studied (*p* > 0.05).

Table 4 shows the multilevel models examining the effects of season, position and half, as well as the effect of an interaction between season and half, on normalised GPS and heart rate variables (model 5). After controlling for these main effects, which showed no major differences from model 4, there was no evidence of a season by half interaction effect within model 5 for any of the dependent variables examined (total distance (*p* = 0.339); high-speed running (*p* = 0.472), accelerations (*p* = 0.652), decelerations (*p* = 0.724), percentage of maximum heart rate (*p* = 0.672) and percentage of time spent at >85% maximum heart rate (*p* = 0.398)). The lack of a cross-level interaction effect between season and half for the six dependent variables is visually presented in Figure 1.

## 4. Discussion

Given recent changes to the structure of matches within the sport of field hockey, the current study sought to determine whether the change in match format from 2 × 35 min halves to 2 × 2 × 17.5 min quarters altered the performance characteristics of male field hockey players across a match and between halves within a match. Using hierarchal linear modelling to account for the nested structure of the data, the study’s analysis suggested that far from eliciting a positive change in the performance characteristics of players as hypothesised, total running distance per half and high-speed running distance per half were reduced by 221 and 120 m, respectively (both *p* < 0.001), when the match format was 2 × 2 × 17.5 min quarters rather than 2 × 35 min halves. The percentage of time spent at >85% of maximum heart rate was also reduced by 3.1% when the match format was 2 × 2 × 17.5 min quarters (*p* = 0.045). In addition, a match format of 2 × 2 × 17.5 min quarters did not attenuate the typically seen decline in performance evident in the second half of matches, as the modelling found no evidence of a season by half interaction effect for any of the performance characteristics examined (total distance (*p* = 0.339); high-speed running (*p* = 0.472), accelerations (*p* = 0.652), decelerations (*p* = 0.724), percentage of maximum heart rate (*p* = 0.672) and percentage of time spent at >85% of maximum heart rate (*p* = 0.398)).

The key rationale for the change in match format (from halves to quarters) initiated by the International Hockey Federation and subsequently adapted by England Hockey is the hypothesis that game “intensity” can be higher when playing bouts are shorter and more numerous. Contrary to that hypothesis, the results from the current study found no evidence that the “quarters” match format had a positive impact on the performance characteristics of male, national-level field hockey players. In fact, running distance covered in total and at high-speed were ~5 and ~14% longer per half when the match was structured as 2 × 35 min periods of competitive play compared with the distances achieved when the match format was 2 × 2 × 17.5 min quarters. Only one other study has performed a comparison similar to that described here. In contrast to the current findings, McMahon and Kennedy [11] found that distances completed in total and at high-speed, in absolute and relative terms (the latter expressing absolute values relative to an appropriate time metric), were always greater when matches were structured in a quarter format in their female international field hockey players. Total distance was 5.1 and 7.5% greater in relative and absolute terms (halves vs. quarters: total running distance, 4880 vs. 5167 m, *d* = 0.2; running distance·min^−1^, 109 vs. 113 m·min^−1^, *d* = 0.6) and high-speed running distance was 4.5 and 0.6% greater (halves vs. quarters: high-speed running distance, 913 vs. 960 m, *d* = 0.1; high-speed running distance·min^−1^, 296 vs. 298 m·min^−1^, *d* = 0.3). An obvious reason for this discrepancy could be that in the study by McMahon and Kennedy [11] the comparison was between 2 × 35 min halves and 2 × 2 × 15 min quarters. However, because the clock was stopped for penalty corners and when goals were scored, McMahon and Kennedy [11] actually found no difference in the time-on-pitch for players when the 2 × 35 min halves and 2 × 2 × 15 min quarters were compared (halves 45.0 ± 9.5 vs. quarters 44.7 ± 11 min, *d* = 0.08). However, their study showed that the relative substitution frequency was 9.4% higher when matches were played in the 2 × 2 × 15 min quarter match format [11]. The use of shorter playing rotations during international competition may have enabled players to work at higher intensities by increasing off-pitch recovery and lowering work: rest ratios. This may be a deliberate strategy in international matches, especially during tournaments where several matches are scheduled within a short timeframe [4]. In comparison, in the current study, there were no stoppages for penalty corners and goals during quarters and the playing time was actually 5% longer when matches were structured as 2 × 2 × 17.5 min playing bouts (halves 48.2 ± 9.4 vs. quarters 50.7 ± 9.9 min). Longer playing rotations and longer time-on-pitch in the male, national-level field hockey players may explain why the performance characteristics examined in the present study were not improved when the match format changed from 2 × 35 min halves to 2 × 2 × 17.5 min quarters. Physiologically, longer playing times would accentuate the accumulation of eccentrically damaging muscle contractions, progressive depletion of muscle glycogen and onset of dehydration that are theorised to explain the reduction in characteristics such as high-speed running distance during field hockey matches [2,22]. Clearly, simply changing the match format to quarters and by implication to shorter playing bouts does not automatically ensure that the performance characteristics demonstrated by players will improve. Given the unlimited substitution rule in field hockey, the number and length of playing bouts in a match are still largely determined by the tactical strategy of the team, and it is the appropriateness of this relative to the performance capabilities of the individual players that may have more influence on the performance characteristics displayed in matches than the specific format of the match.

In the current study, the change in match format from 2 × 35 min halves to 2 × 2 × 17.5 min quarters also resulted in a 3.1% reduction in the percentage of time spent at >85% of maximum heart rate in a half. The introduction of 2 min quarter time intervals has been proposed to facilitate recovery in field hockey players [13]. If lower/similar heart rate responses are seen together with similar/improved performance characteristics, a performance and/or physiological benefit of the halves to quarter match format rule change could be implied [23]. However, in the present study, the 3.1% reduction in the percentage of time spent at >85% of maximum heart rate in a half when the match format was 2 × 2 × 17.5 min quarters was seen alongside a reduction in total distance and high-speed running completed during a match (by 221 m and 120 m, respectively, both *p* < 0.001). Therefore, despite the theoretical benefits on recovery and performance characteristics of a match format based on quarters, a number of performance characteristics of the players in the current study actually declined. It may be that the university players examined here were already performing at their limits before the change in match format was implemented, and while structurally a quarter format might in principle allow improved performance, this is only possible if the players’ capabilities are not already near or at maximum. Studying a comparable age demographic to that in the present study (21.2 ± 3.0 vs. 21.1 ± 3.4 years), Sunderland and Edwards [16] used similar reasoning to explain why there were no differences in total distance or high-speed running distance performed by UK national-level players when the self-pass rule was introduced. It has also been suggested that the superior performance capabilities of higher standard players (particularly internationals) may allow international standard players to perform and sustain more high-speed running distance compared to national-level players and hence take advantage of the recovery opportunities created when match format is four quarters rather than two halves [7,11]. Again, it would appear that manipulation of match format alone does not ensure that the performance characteristics of field hockey players during competition will be improved.

Another issue examined in the current study related to the typically seen reduction in performance characteristics evident during the second half of field hockey matches organised in 2 × 35 min periods (this first to second half decline is also evident in many other team sports such as association football and rugby union; [24,25]). Some recent studies in field hockey have found that a key performance characteristic such as high-speed running distance does not obviously decline when the match format is 4 × 15 min [13,14,26], which would support the reasoning underpinning the match format change first implemented by the International Hockey Federation in 2014. However, none of these studies directly compared the two types of match format, and in the current study (which did make this direct comparison), total and high-speed running distances were less, and the number of accelerations and decelerations were fewer in the second half of matches, regardless of whether the match format was 2 × 35 min or 2 × 2 × 17.5 min. In addition, there was no evidence of an interaction effect between season and half in the multilevel modelling for any of the performance characteristics studied (Table 4, Figure 1), so the pattern of change from the first to second half of matches appeared to be similar for both match formats examined in the current study. These findings suggest that a match format where competitive playing bouts are shorter and more numerous does not necessarily attenuate the reduction in performance typically seen during the second half of field hockey matches.

The inclusion of position as an explanatory variable within the multilevel models fitted in the current study suggested that defenders covered less total distance (by −632/−734 m) and less high-speed running distance (by −429/−633 m), and performed fewer accelerations (by −19/−25) and decelerations (by −20/−24) in a half of match play compared to midfielders and forwards, respectively (all *p* < 0.001; Table 3). Forwards also covered more high-speed running distance (*p* < 0.001), and completed a greater number of accelerations (*p* < 0.001) and decelerations (*p* = 0.027) in a half of match play than midfielders. No inter-positional differences were evident for the percentage of maximum heart rate maintained during a half. These observed differences in performance characteristics based on playing position are consistent with most other studies in the research literature, which have also noted that the values of various performance characteristics are lower in defenders than those exhibited by players in midfield and forward positions [6,7,16].

It is important to note the limitations of the current study. The data presented are from one team and may not be representative of other national league teams. The physical demands could have been influenced by differences in the tactical approach to individual games, such as substitution strategy. Contextual issues such as the strength of opposition and number of goals scored and conceded may also have influenced the results; however, as data were collected over two whole seasons this is unlikely to have had a large influence on the overall results of the study.

## 5. Conclusions

A key rationale for changes in match format (from halves to quarters) initiated by the International Hockey Federation, and subsequently adapted by England Hockey, is the hypothesis that game intensity can be higher when competitive playing bouts are shorter and more numerous. The current study sought to determine whether a change in match format from 2 × 35 min halves to 2 × 2 × 17.5 min quarters altered the performance characteristics of male field hockey players across a match and between halves within a match. Overall, the findings from the current study suggest that the change in match structure did alter the performance characteristics of male university field hockey players, but the quarter format actually reduced the total distance and high-speed running distance completed during matches, and did not attenuate the reduction in performance seen during the second half of matches.

## Figures and Tables

**Table 1:** Performance characteristics (descriptive data) of male university field hockey players during 70 min national-level matches, when the match format was 2 × 35 min halves (2018–2019 season) or 2 × 2 × 17.5 min quarters (2019–2020 season). Global Positioning System (GPS) and heart rate variables are presented as mean ± SD.

	Mean ± SD	
Performance Characteristic	2018–2019 (2 × 35 min Halves)	2019–2020 (2 × 2 × 17.5 min Quarters)
Time on pitch (min)	48.2 ± 9.4	50.7 ± 9.9
Total distance (m)	6424 ± 893	6456 ± 820
Distance per minute (m·min^−1^)	135 ± 15	130 ± 15
High-speed running (m)	1485 ± 412	1434 ± 433
High-speed running% (%total distance)	20.6 ± 5.6	18.6 ± 5.4
Accelerations (n)	75 ± 16	78 ± 18
Accelerations per minute (n·min^−1^)	1.61 ± 0.45	1.59 ± 0.46
Decelerations (n)	76 ± 15	81 ± 18
Decelerations per minute (n·min^−1^)	1.62 ± 0.40	1.66 ± 0.47
HR_AVE_ (b·min^−1^)	173 ± 8	168 ± 11
%Maximum heart rate	85.9 ± 3.6	83.4 ± 4.6
HR_PEAK_ (b·min^−1^)	195 ± 6	193 ± 8
HR_PEAK_ (%Maximum heart rate)	96.9 ± 2.0	96.2 ± 2.3
Time spent > 85% HR_MAX_ (min)	32.8 ± 10.9	28.0 ± 10.8
Percentage time > 85% HR_MAX_ (%)	67.4 ± 16.1	56.1 ± 20.6

HR_AVE_ = average heart rate during match; HR_PEAK_ = peak heart rate during match; Time spent > 85% HR_MAX_ (min) = duration (min) of total time spent at a zone exceeding 85% of maximum heart rate; Percentage time > 85% HR_MAX_ (%) = percentage (%) of total time spent at a zone exceeding 85% of maximum heart rate.

**Table 2:** Performance characteristics (descriptive data) of male university field hockey players across 2 × 2 × 17.5 min quarters during 70 min national-level matches played in the 2019–2020 season. Global Positioning System (GPS) and heart rate variables are presented as mean ± SD.

	Mean ± SD			
Performance Characteristic	1st Quarter	2nd Quarter	3rd Quarter	4th Quarter
Time on pitch (min)	12.5 ± 3.3	12.8 ± 3.2	12.9 ± 3.7	12.7 ± 3.5
Total distance (m)	1652 ± 339	1643 ± 344	1573 ± 368	1587 ± 402
Distance per minute (m·min^−1^)	135 ± 19	131 ± 17	125 ± 19	127 ± 19
High-speed running (m)	364 ± 139	356 ± 140	342 ± 138	347 ± 148
High-speed running% (%total distance)	22.4 ± 7.7	21.7 ± 7.0	22.0 ± 7.4	22.0 ± 7.6
Accelerations (n)	20 ± 7	19 ± 7	19 ± 7	19 ± 7
Accelerations per minute (n·min^−1^)	1.70 ± 0.57	1.58 ± 0.57	1.52 ± 0.53	1.52 ± 0.53
Decelerations (n)	21 ± 6	20 ± 7	19 ± 7	19 ± 7
Decelerations per minute (n·min^−1^)	1.77 ± 0.58	1.65 ± 0.56	1.56 ± 0.55	1.59 ± 0.56
HR_AVE_ (b·min^−1^)	170 ± 12	170 ± 12	166 ± 13	166 ± 14
%Maximum heart rate	84.5 ± 5.2	84.5 ± 5.1	82.3 ± 5.9	82.4 ± 6.0
HR_PEAK_ (b·min^−1^)	191 ± 9	191 ± 8	188 ± 9	186 ± 11
HR_PEAK_ (%Maximum heart rate)	94.9 ± 3.0	94.9 ± 2.6	93.7 ± 3.6	92.5 ± 4.3
Time spent > 85% HR_MAX_ (min)	7.5 ± 3.3	7.7 ± 3.5	6.4 ± 3.3	6.4 ± 3.4
Percentage time > 85% HR_MAX_ (%)	60.8 ± 23.8	60.8 ± 23.5	51.1 ± 24.2	51.0 ± 24.4

HR_AVE_ = average heart rate during match; HR_PEAK_ = peak heart rate during match; Time spent > 85% HR_MAX_ (min) = duration (min) of total time spent at a zone exceeding 85% of maximum heart rate; Percentage time > 85% HR_MAX_ (%) = percentage (%) of total time spent at a zone exceeding 85% of maximum heart rate.

**Table 3:** Multilevel models examining the effects of season, position and half on normalised Global Positioning System (GPS) and heart rate variables (model 4), using 1st and 2nd half data, from matches where the playing format was 2 × 35 min halves (2018–2019 season) or 2 × 2 × 17.5 min quarters (2019–2020 season).

Dependent Variable		Total Distance (m)	High-Speed Running Distance (m)	Accelerations (n)	Decelerations (n)	%Maximum Heart Rate	Percentage of time spent at >85% HR_MAX_ (%)
		**Mean (SE)**					
*Fixed Component*							
Baseline	Intercept	4415 (92)	830 (61)	45 (3)	46 (3)	84.2 (1.5)	63.4 (4.8)
Season (Ref: 2018–2019)	2019–2020	−221 (30) *	−120 (19) *	−1 (1)	−1 (1)	−0.6 (0.4)	−3.1 (1.5) *
Position (Ref: Defenders)	Midfielders	+632 (110) ^a^	+429 (73) ^a^	+19 (4) ^a^	+20 (3) ^a^	+2.0 (1.8)	+6.4 (5.8)
	Forwards	+734 (109) ^a^	+633 (72) ^ab^	+25 (4) ^ab^	+24 (3) ^ab^	+1.5 (1.7)	+6.3 (5.7)
Half (Ref: 1st Half)	2nd Half	−235 (19) ^+^	−70 (12) ^+^	−4 (1) ^+^	−4 (1) ^+^	−2.2 (0.2) ^+^	−10.3 (0.9) ^+^
*Random Component*							
Between-player SD		272	183	10	8	4.5	14.4
Between-match file SD		323	203	9	9	3.6	15.3
Δ Deviance	Model 1 to Model 2 (Baseline + Season)	54 ^ɫ^	55 ^ɫ^	4.3 ^ɫ^	3.0	2.4	3.7
	Model 2 to Model 3 (Model 2 + Position)	30 ^ɫ^	63 ^ɫ^	35 ^ɫ^	33 ^ɫ^	1.4	1.3
	Model 3 to Model 4 (Model 3 + Half)	150 ^ɫ^	36 ^ɫ^	59 ^ɫ^	65 ^ɫ^	97^ɫ^	133 ^ɫ^

Significance = *p* < 0.05. * = Significant difference from 2018–2019 season. ^a^ = Significant difference from defenders. ^b^ = Significant difference from midfielders. ^+^ = Significant difference from 1st half. ^ɫ^ = Significantly better model fit. SD—standard deviation (√variance). Dependent variables were normalised by extrapolating values to the full half playing time of 35 min. Percentage of time spent at >85% HR_MAX_ = percentage (%) of total time spent at a zone exceeding 85% of maximum heart rate.

**Table 4:** Multilevel models examining the effects of season, position and half, and a cross-level interaction between season and half on normalised Global Positioning System (GPS) and heart rate variables (model 5), using 1st and 2nd half data, from matches where the playing format was 2 × 35 min halves (2018–2019 season) or 2 × 2 × 17.5 min quarters (2019–2020 season).

Dependent Variable		Total Distance (m)	High-Speed Running Distance (m)	Accelerations (n)	Decelerations (n)	%Maximum Heart Rate	Percentage of Time Spent at >85% HR_MAX_ (%)
		**Mean (SE)**					
Fixed Component							
Baseline	Intercept	4404 (92)	835 (61)	45 (3)	46 (3)	84.3 (1.5)	63.9 (4.9)
Season (Ref: 2018–2019)	2019–2020	−202 (35) *	−128 (22) *	−1 (1)	−1 (1)	−0.7 (0.4)	−3.9 (1.8) *
Position (Ref: Defenders)	Midfielders	+632 (110) ^a^	+429 (73) ^a^	+19 (4) ^a^	+20 (3) ^a^	+2.0 (1.8)	+6.4 (5.8)
	Forwards	+734 (109) ^a^	+633 (72) ^ab^	+25 (4) ^ab^	+24 (3) ^ab^	+1.5 (1.7)	+6.3 (5.7)
Half (Ref: 1st Half)	2nd Half	−214 (29) ^+^	−80 (18) ^+^	−4 (1) ^+^	−4 (1) ^+^	−2.4 (0.4) ^+^	−11.3 (1.5) ^+^
Season*Half (Ref: 2018–2019*1st Half)	2019–2020*2nd Half	−36 (38)	+17 (24)	0 (1)	0 (1)	+0.2 (0.5)	+1.6 (1.9)
Random Component							
Between-player SD		272	183	10	8	4.5	14.4
Between-match file SD		323	203	9	9	3.6	15.3
Δ Deviance	Model 4 to Model 5 (Model 4 + Season*Half)	0.9	0.5	0.7	0.1	0.2	0.5

Significance = *p* < 0.05. * = Significant difference from 2018–2019 season. ^a^ = Significant difference from defenders. ^b^ = Significant difference from midfielders. ^+^ = Significant difference from 1st half. SD—standard deviation (√variance). Dependent variables were normalised by extrapolating values to the full half playing time of 35 min. Percentage of time spent at >85% HR_MAX_ = percentage (%) of total time spent at a zone exceeding 85% of maximum heart rate.

## Data Availability

The data presented in this study are available on request from the corresponding author.
